# Supplementary material for: Coronatine Enhances Chilling Tolerance of Tomato Plants by Inducing Chilling-Related Epigenetic Adaptations and Transcriptional Reprogramming
Source: Int J Mol Sci. 2022 Sep 2;23(17):10049. doi: 10.3390/ijms231710049 (PMC9456409; doi:10.3390/ijms231710049)
Supplement: Supplementary file 1 [file ijms-23-10049-s001.zip › SUPPLEMENTAL FIGURES.pptx]

## Slide 1
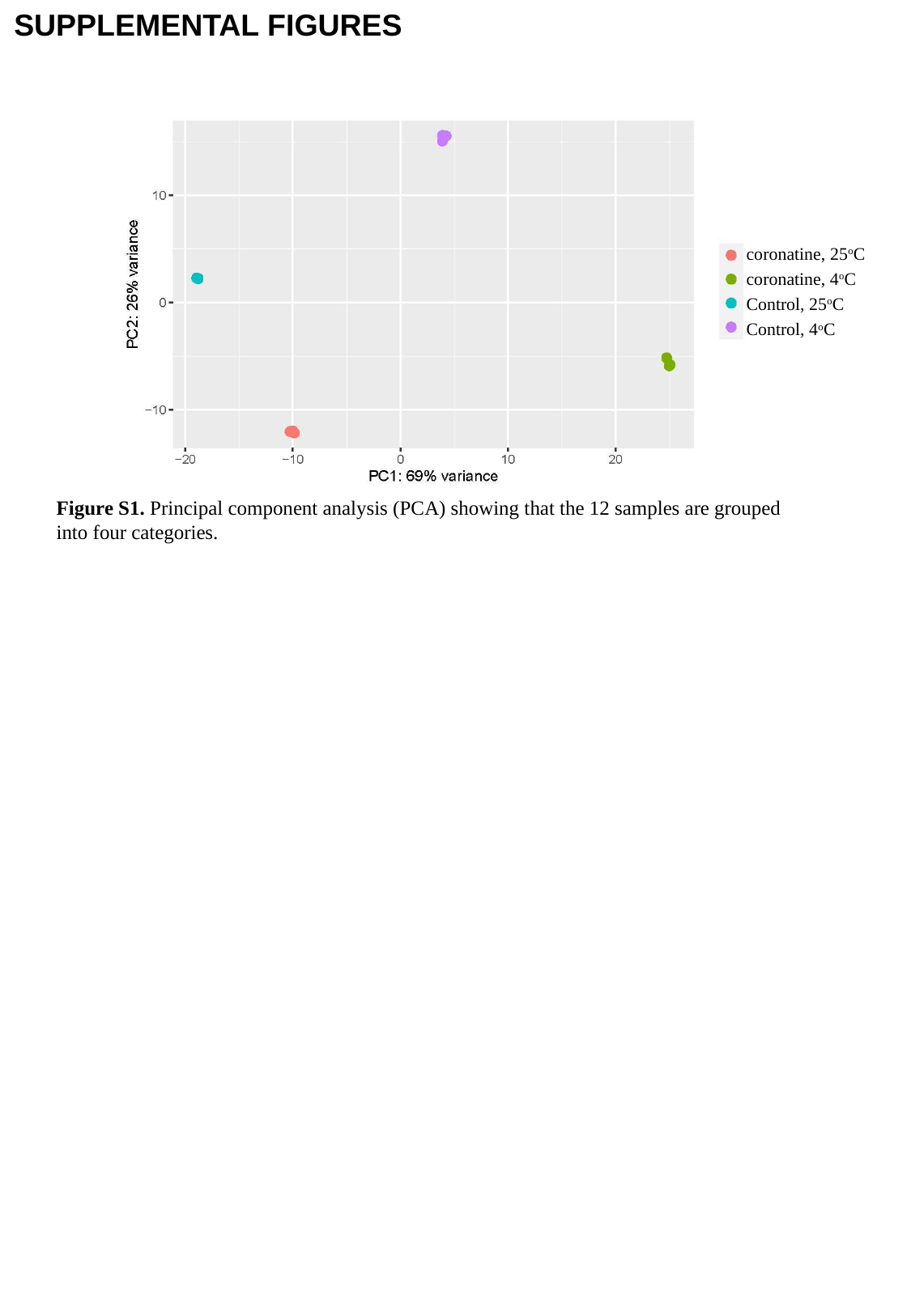

SUPPLEMENTAL FIGURES
coronatine, 25oC
coronatine, 4oC
Control, 25oC
Control, 4oC
Figure S1. Principal component analysis (PCA) showing that the 12 samples are grouped into four categories.

## Slide 2
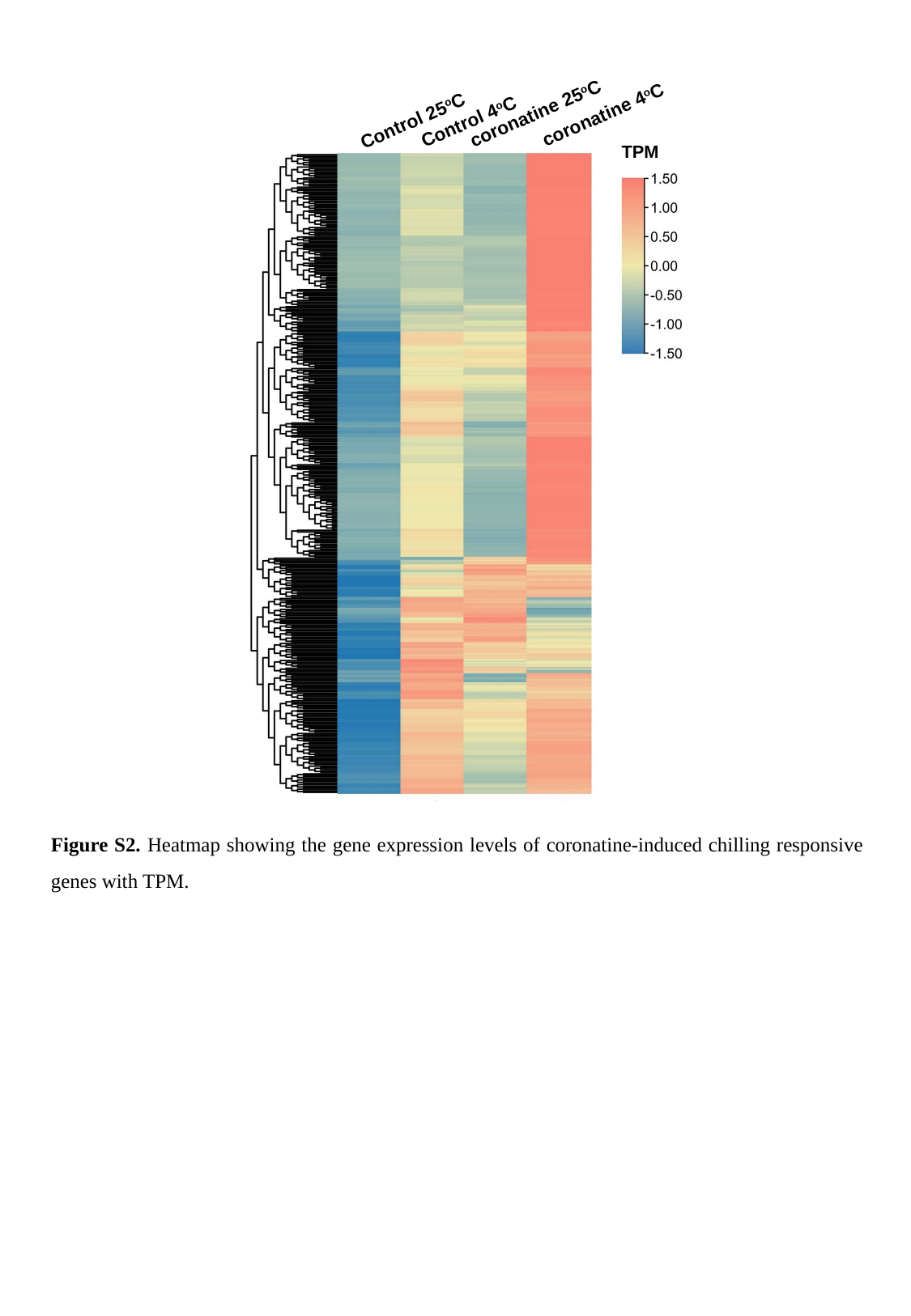

coronatine 25oC
coronatine 4oC
Control 25oC
Control 4oC
TPM
Figure S2. Heatmap showing the gene expression levels of coronatine-induced chilling responsive genes with TPM.

## Slide 3
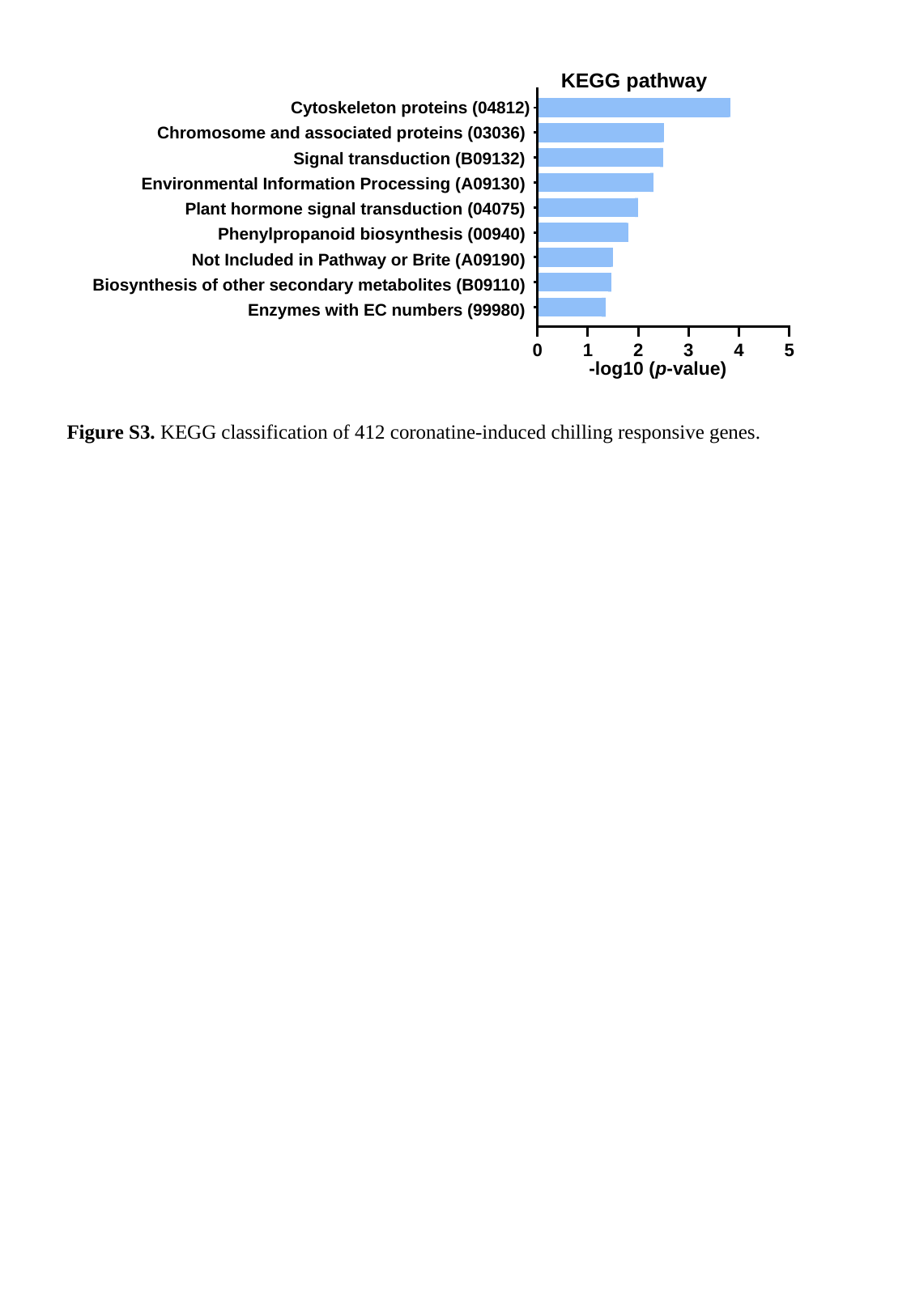

KEGG pathway
Cytoskeleton proteins (04812)
Chromosome and associated proteins (03036)
Signal transduction (B09132)
Environmental Information Processing (A09130)
Plant hormone signal transduction (04075)
Phenylpropanoid biosynthesis (00940)
Not Included in Pathway or Brite (A09190)
Biosynthesis of other secondary metabolites (B09110)
Enzymes with EC numbers (99980)
-log10 (p-value)
Figure S3. KEGG classification of 412 coronatine-induced chilling responsive genes.

## Slide 4
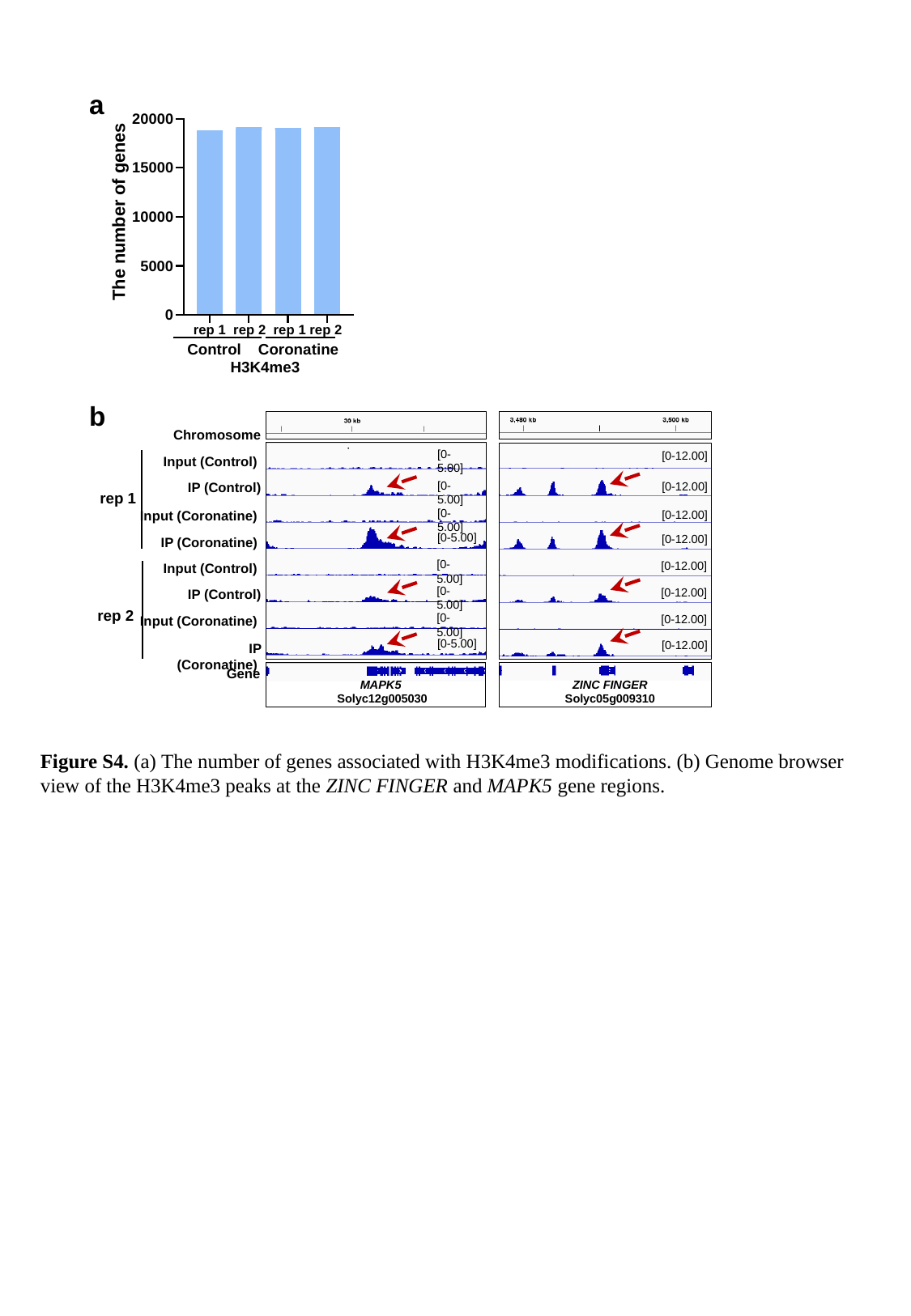

a
The number of genes
rep 1 rep 2 rep 1 rep 2
Control Coronatine
H3K4me3
b
Chromosome
[0-5.00]
[0-12.00]
Input (Control)
[0-5.00]
IP (Control)
[0-12.00]
rep 1
[0-5.00]
Input (Coronatine)
[0-12.00]
[0-5.00]
[0-12.00]
IP (Coronatine)
[0-5.00]
[0-12.00]
Input (Control)
[0-5.00]
[0-12.00]
IP (Control)
rep 2
[0-5.00]
[0-12.00]
Input (Coronatine)
[0-5.00]
[0-12.00]
IP (Coronatine)
Gene
ZINC FINGER
Solyc05g009310
MAPK5
Solyc12g005030
Figure S4. (a) The number of genes associated with H3K4me3 modifications. (b) Genome browser view of the H3K4me3 peaks at the ZINC FINGER and MAPK5 gene regions.
