# Supplementary material for: Coronatine Enhances Chilling Tolerance of Tomato Plants by Inducing Chilling-Related Epigenetic Adaptations and Transcriptional Reprogramming
Source: Int J Mol Sci. 2022 Sep 2;23(17):10049. doi: 10.3390/ijms231710049 (PMC9456409; doi:10.3390/ijms231710049)
Supplement: Supplementary file 1 [file ijms-23-10049-s001.zip › Supplementary table S10.pdf]

Supplemental Table S10, Overview of the primers used for RT-qPCR.

**Primers for quantitative real-time PCR**	
PI-II-qRT-F	AATTATCCATCATGGCTGTTCAC
PI-II-qRT-R	CCTTTTTGGATCAGATTCTCCTT
TD-qRT-F	AGCTCAAACACACGCGCTGGA
TD-qRT-R	AACCCCCACCACCAACAGGT
PR1-qRT-F	ATCTCATTGTTACTCACTTGTC
PR1-qRT-R	AACGAGCCCGACCA
SlCBF1-F	GACTTCGTGGATGAGGAGGC
SlCBF1-R	CTGCACATTGAGGTGGAGGT
SlCBF2-F	GGCTGCCTACTCCAGATTCC
SlCBF2-R	ACCACTGATTCTTCTTCCTCCG
SlCBF3-F	ACTTCGTGGATGAGGAAGCG
SlCBF3-R	GGCATATAAGCGTGCACATCA
ACT2-F	TTGCTGACCGTATGAGCAAG
ACT2-R	GGACAATGGATGGACCAGAC
WRKY33-F	GAATTGGGGATTTAGCGAGGA
WRKY33-R	TGTTGAACAAAACGGGCGAG
WRKY39-F	TTAGCAGCCGCCATATCAGG
WRKY39-R	TGGCTGCCTGTCTTTCTCTG
ZF14-F	TGCCACTAGTGATTGGCAGG
ZF14-R	AAATGGCACATTTTGCGGCT
ZF (Solyc03g006250)-F	TATCGCGGAGGGTATGGGAT
ZF (Solyc03g006250)-R	TGCTCCTGCCACTGCAAAAT
